# *Aedes aegypti* spreading in South America: new coldest and southernmost records

**DOI:** 10.1590/0074-02760190496

**Published:** 2020-05-08

**Authors:** Alejandra Rubio, María Victoria Cardo, Darío Vezzani, Aníbal Eduardo Carbajo

**Affiliations:** 1 Consejo Nacional de Investigaciones Científicas y Técnicas, Ciudad Autónoma de Buenos Aires, Argentina; 2 Universidad Nacional de San Martín, Consejo Nacional de Investigaciones Científicas y Técnicas, Instituto de Investigación e Ingeniería Ambiental, Ecología de Enfermedades Transmitidas por Vectores, San Martín, Provincia de Buenos Aires, Argentina; 3 Universidad Nacional del Centro de la Provincia de Buenos Aires, Facultad de Ciencias Exactas, Instituto Multidisciplinario sobre Ecosistemas y Desarrollo Sustentable, Tandil, Provincia de Buenos Aires, Argentina

**Keywords:** dengue vector, South American distribution, extreme environmental conditions, Patagonia.

## Abstract

The geographic distribution of *Aedes (Stegomyia) aegypti* (L.) in South America has been expanding during the last decades. Herein we present two new distribution records that extend its southern limits towards localities with extremer environmental conditions than reported to date. San Antonio Oeste constitutes the southernmost finding for the continent (40º44’S), whereas Tandil is the infested locality with the coldest mean annual temperature in Argentina (14.17ºC). The projection of a previous distribution model for *Ae. aegypti* predicts these two cities as positive and suggests several other localities with suitable conditions for vector proliferation beyond its assumed distribution limits.

Over half of the world’s population live in areas where *Aedes (Stegomyia) aegypti* (L.), the main vector of dengue, chikungunya, Zika and yellow fever, occurs.^1^ ^,^ ^2^ This species is the only member of the genus present in artificial containers along the South American southern fringe.^3^ The expansion patterns of *Ae. aegypti* have been mainly assessed using discarded tires and cemetery flower vases as strategic containers due to their homogeneous characteristics that make them suitable for comparative purposes,^3^ ^,^ ^4^ ^,^ ^5^ but fortuitous findings in residential containers have also provided valuable new records.^6^ Passive dispersion through human transportation has been postulated as a main driver in the colonisation of urban settings at higher latitudes,^7^ mechanism in which discarded tires on highways play a key role.^8^

The distribution limit of *Ae. aegypti* in South America has been in constant expansion from tropical to temperate regions since the continental eradication program was discontinued in the 1970s. Toward the year 2000, the southern distribution fringe crossed Argentina from the northwest to the southeast, with positive localities at latitudes up to 35ºS.^9^ During the last decade, the distribution has expanded further towards the west and south following a pattern of isolated new records that rarely exceed 37ºS. Exceptions to this pattern are the two so far southernmost records at 38ºS in urban agglomerates with around 300,000 inhabitants, Neuquén City in Neuquén Province to the west and Bahía Blanca in Buenos Aires Province to the east.^3^ ^,^ ^10^

The current geographic distribution of *Ae. aegypti* at the global scale and its potential spread under different climate change scenarios have been modelled integrating climatic conditions and anthropogenic factors,^11^ but these models generally fail to predict vector presence at the limits of its distribution. In the South American fringe, a regional model suggested that the past and present distributions of *Ae. aegypti* were associated with a quantitative compromise between air temperature and human population number.^3^ Herein, we update the distribution limit of *Ae. aegypti* in South America including the southernmost and coldest records for the species. We also analyse these new findings in the context of the regional distribution model mentioned above.

A mosquito survey was performed in 15 localities without previous records of *Ae. aegypti* from 37 to 47ºS encompassing southern Buenos Aires Province and eastern Patagonia, Argentina. Mean annual temperature ranges from 16.5ºC to 11ºC,^12^ and isotherms present a non-flat pattern as the result of the joint interaction of topography and oceanic influence (Figure). East Patagonia is a vast and very dry region, where dwellings are spaced by hundreds of kilometres. Therefore, fuel stations are the obligate stop for travellers and frequently constitute the only available source of water, shelter and shade, representing a suitable habitat for mosquitoes. In January 2019 an active search for mosquitoes was carried out by inspecting tire-repair shops, fuel stations and cemeteries along main roads across the southwest of Buenos Aires Province, the east of La Pampa, Río Negro and Chubut provinces, and the northeast of Santa Cruz Province. The surveillance included 14 localities along the Provincial Route Nº 51 and National Routes Nº 22, 251 and 3. During February and March 2019, residential premises, tire-shops and abandoned tires distributed throughout Tandil, at the southeast of Buenos Aires Province, were inspected. At each site, water holding containers were searched for immatures with a fine mesh net and adults were collected with manual or battery-powered aspirators. Larvae were fixed in 70% ethanol and pupae were reared to adults which were kept in freezer for further identification.

Recently, Carbajo et al.^3^ proposed a model for the occurrence of *Ae. aegypti* in Buenos Aires and La Pampa provinces at the locality scale. In the light of our new records, we extended the projections of such model to Neuquén, Río Negro, Chubut and Santa Cruz provinces. For each locality, a lineal predictor (LP) was calculated as

LP = − 0.09 + 2.68 x Temp + 1.97 x Logpo

where Temp is the mean annual temperature obtained by averaging the 6 years’ period 2013-2018 based on monthly data from CRU TS4.03,^12^ standardised with mean and standard deviation of the original model temperature (15.67 and 1.05, respectively); and Logpo is the logarithm of the total population projected for 2018,^13^ standardised likewise (9.46 and 1.35). To obtain a probability, the LP was logit transformed as exp (LP)/(1 + exp (LP)); a locality was considered positive if its probability value exceeded the optimum cut-off point of the original model (0.29). Spatial data processing was performed with QGIS 2.10.1 Pisa.

Out of 15 localities inspected (Table, Figure), new records are reported for two of them, namely San Antonio Oeste (19,821 inhabitants, 40º43’55”S - 64º56’52”W, Río Negro Province) and Tandil (133,622 inhabitants, 37º19′08″S - 59º08′05″W, Buenos Aires Province). The former constitutes the southernmost record of *Ae. aegypti* in South America (Figure, locality D), whereas the latter is the positive locality with the coldest mean annual temperature in Argentina (14.17ºC in the period 2013-2018) (Figure, locality A).

**TABLE Sites:** sampled by locality, ordered from north to south

Locality (Province)	Sampled sites
*Tandil (BA)	nine dwellings, three tire repair shops, 24 abandoned tires
Coronel Pringles (BA)	one tire repair shop, one public toilet
Médanos (BA)	two tire repair shops, two public toilets
La Adela (LP)	one public toilet
Río Colorado (RN)	one tire repair shop, one public toilet
General Conesa (RN)	two public toilets
*San Antonio Oeste (RN)	one tire repair shop, two public toilets
Sierra Grande (RN)	one tire repair shop, one public toilet
Puerto Madryn (CH)	three public toilets, one cemetery
Trelew (CH)	three public toilets
Gaiman (CH)	one cemetery
Garayalde (CH)	one public toilet
Comodoro Rivadavia (CH)	two repair shops, three public toilets, one cemetery
Caleta Olivia (SC)	one cemetery, two public toilets
Fitz Roy (SC)	one tire repair shop, two public toilets

*: positive locality; BA: Buenos Aires; LP: La Pampa; RN: Río Negro; CH: Chubut; SC: Santa Cruz.

**Figure:**
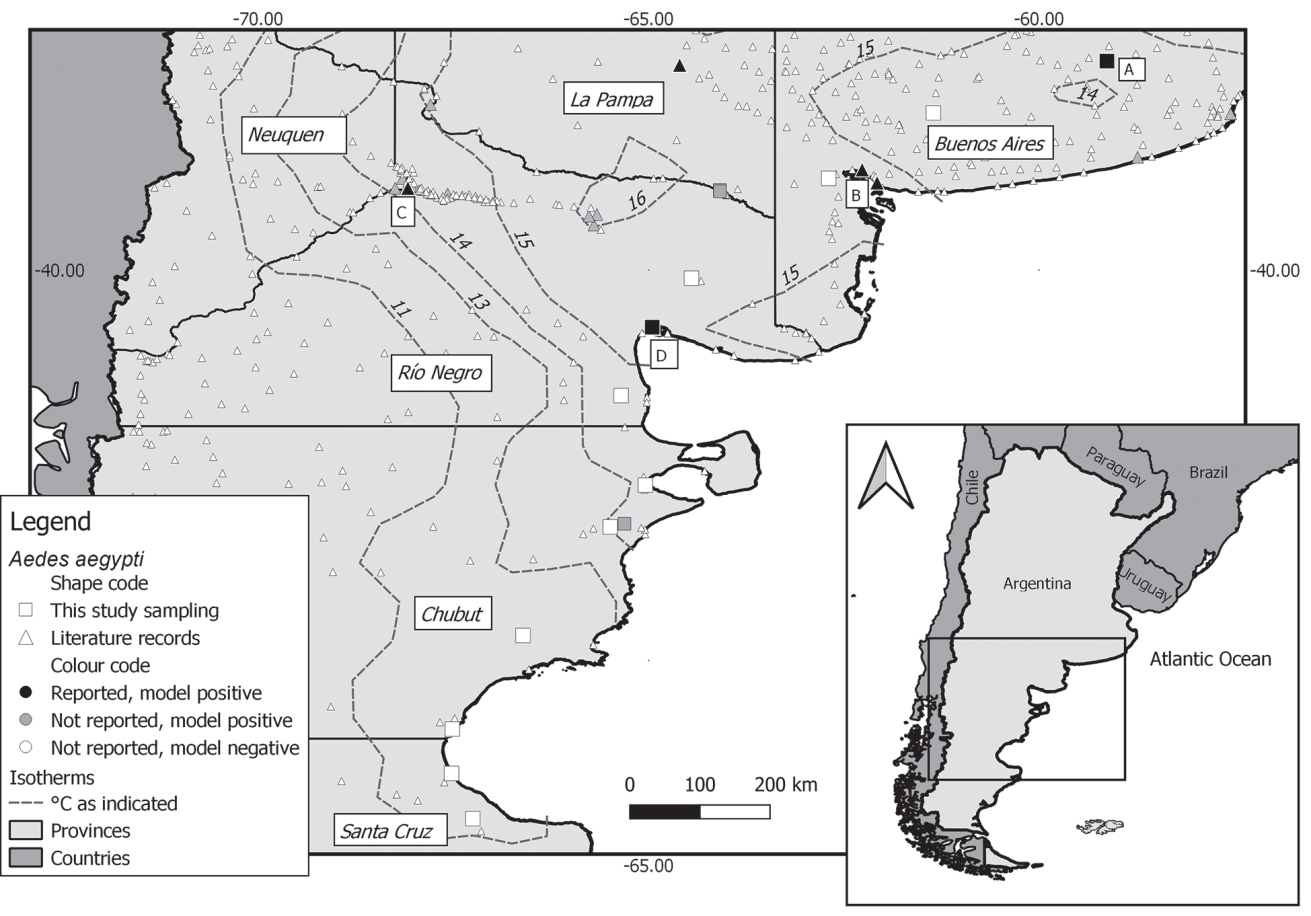
Occurrence of *Aedes aegypti* in localities of the study area. Letters in the map indicate cities mentioned in the text: (A) Tandil; (B) Bahía Blanca and Punta Alta; (C) Neuquén; (D) San Antonio Oeste.

In San Antonio Oeste, three adults of *Ae. aegypti* (one female, two males) were obtained from pupae collected in an uncovered water-tank placed at ground level in a tire-repair shop on the outskirts of the city. In Tandil, eight adults (three females, five males) were captured in four private premises, whereas 35 immatures were found in other two dwellings (20 individuals in a plastic bucket used as base for a flower pot and 15 in a canvas fold).

The model for the occurrence of *Ae. aegypti* projected for Neuquén, Río Negro, Chubut and Santa Cruz provinces showed 12 localities up to 43ºS with suitable climatic and demographic conditions to be positive for the vector (Figure). Among these localities are Neuquén City (Figure, locality C), in which vector presence was recorded in 2010,^10^ and San Antonio Oeste (this study). In Buenos Aires Province, the model predicts favourable conditions for the occurrence of *Ae. aegypti* in four localities south of Tandil, including Bahía Blanca and neighbouring Punta Alta (Figure, localities B) in which it has already been reported.^3^

The new records presented herein extend the South American southern occurrence limits of *Ae. aegypti* towards more extreme localities both in terms of geographical location and climatic conditions than known to date. Our survey reports the presence of this mosquito almost 200 km south than previous southernmost records, whereas Tandil is the positive city with lowest mean annual temperature in Argentina. Although the finding of immatures and adults at several residential premises in different areas of Tandil strongly suggests that a population of the vector is established, this could not be guaranteed in localities next to the distribution limit. It is presumed that, during the warm season, specimens of *Ae. aegypti* colonise areas that do not support a year-round population.^4^ ^,^ ^10^ For example, the vector was reported in Neuquén City in 2010^10^ but no eggs were collected during a weekly search by ovitraps during January-April 2016.^14^ For the samples collected in San Antonio Oeste, however, it is unlikely that these eggs had been transported from another locality because at the inspected tire shop the water came directly from the drinking water network and the tank was not used as a tire washing bathtub. However, it is feasible that these individuals came from eggs laid by gravid females which were introduced to the locality by travel or commerce transport in the same breeding season. If populations are established at such high latitudes is a matter of further research.

During the last decade, *Ae. aegypti* has been reported in areas which were previously considered unsuitable in terms of climatic conditions. There, urbanisation features and other socioeconomic factors may play a key role in defining apt habitat.^7^ Also, Carbajo et al.^3^ showed that mean annual isotherms are shifting south and west in temperate Argentina, progressively enlarging the climatic suitable region for the development of *Ae. aegypti*. The distribution model for the expected occurrence of the vector in central and southern Argentina highlights several localities with potentially favorable conditions that were not predicted by large-scale models.^7^ ^,^ ^15^ If vector occurrence is determined by a compromise between air temperature and human population size, restricting temperatures for the completion of mosquito life cycle could be compensated by the use of urban features as shelter and food at the microscale, as well as inherent urban processes such as the heat island effect at meso scales.^3^ Notwithstanding, the present analysis is not intended to validate the model in Carbajo et al.,^3^ as it is an extrapolation to an untrained area. Also, sampling at certain localities was restricted to a few sites, this was the case of two localities for which the model predicted the occurrence of *Ae. aegypti* but no specimens were collected. Intensive sampling in land uses typically associated with the vector, such as dwellings, is encouraged for such localities. However, this fact does not undermine the strength of the positive records presented, rather the contrary, as the occurrence of *Ae. aegypti* in San Antonio Oeste was documented with a low sampling effort.

Pressure on filling unoccupied habitats will inevitably lead to the establishment of permanent populations of *Ae. aegypti* in the majority of the localities where conditions for their survival are met.^7^ In the South American southern cone, it is clear that the vector continues to expand southwards to localities in which it is able to complete its life cycle at least during one season. Whether this is a physiological adaptation of certain mosquito strains to cooler areas mediated by photoperiod-induced embryonic dormancy^16^ or a result of a south shift of the isotherms due to climate change remains unanswered. In accordance with Kraemer et al.,^7^ who argue that global modeled rates of spread are underestimated because global datasets of records of *Ae. aegypti* are spatio-temporally biased, we encourage routine and systematic surveillance for vector presence especially in the margins of its distribution. The breakneck expansion of *Ae. aegypti* to unprecedented latitudes in South America alerts on the need to implement constant entomological surveillance for disease transmitting mosquitoes far beyond its assumed distribution limits. Theoretical tools, such as the implementation of predictive models for mosquito presence, can help optimise vector surveillance efforts.
